# Seasonal pattern of vitamin D in male elite soccer players

**DOI:** 10.1186/1550-2783-8-S1-P35

**Published:** 2011-11-07

**Authors:** Fabrizio Angelini, Fulvio Marzatico, Gianluca Stesina, Luca Stefanini, Alessandro Bonuccelli, Stefano Beschi, Daniela Buonocore, Sara Rucci, Fabrizio Tencone

**Affiliations:** 1 Society of Sport Nutrition and Wellness; 2 Medical Staff, Juventus FC, Turin, Italy; 3 Laboratory of Pharmacobiochemistry, Nutrition and Nutriceuticals of Health, University of Pavia, Pavia, Italy

## Background

The phopho-calcium metabolism and the maintenance of bone mass is not the only important role vitamin Dplays. Vitamin D is also known for its anti-inflammatory function and for modulating the immune defence system. The vitamin D deficit is to be referred not simply to a bone tissue worsening, but to cardiovascular diseases, various types of tumours and some autoimmune diseases. In the sport life, a vitamin D deficit is often related to muscular problems, neuromuscular pains, predisposition to injuries, and can affect one’s performance. Since indoor athletes have reduced exposition to sun rays, they are more likely to be subjected to these risks than outdoor athletes. However, in soccer, the athletes can experience vitamin D deficit not just during the winter but in other periods too, most likely due to several reasons such as, dark complexion, coming from high altitude championships, injuries, or inadequate exposition to sun rays during the summer. The purpose of this study was to examine the vitamin D shortage and BMC variations in Italian Serie A elite male soccer players.

## Methods

The BMC was measured with DXA methodology (Hologic QDR-4500A) at the end of the summer season and during the winter while the concentration of 25 (OH) vitamin D (25(OH)D3) was registered in twenty-three athletes of 28.1 ± 4.8 of age (Average ± DS) during a whole soccer season by means of three samplings, one at the end of the summer season, one during the winter season and one in spring.

## Results

The concentration of 25(OH) D3was 111.5± 30.5, 92.3 ± 30.8 and 102.5±37.1 nmol/L (Average ± DS) in autumn, winter and spring respectively. The concentration of 25(OH)D3 significantly decreased from autumn to winter (P<0.001) while no differences were registered in other seasons comparisons (P>0.05). Using: a) concentrations of 100 nmol/L as optimal cut-off, 40.9 %, 56.0 % e 52.0 % players had sub-optimal levels of 25(OH)D3 in autumn, winter and spring respectively, b) concentrations < di 80 nmol/L ma > of 50 nmol/L as an index of shortage, 9.1 %, 32.0 % e 28.0 % players had insufficient 25(OH)D3 levels in autumn, winter and spring respectively, c) concentrations ≤ a 50 nmol/L as an index of shortage, the percentage of soccer player in shortage of vitamin D was nearly doubled between winter and autumn, from 4.5 % to 8.0%, then reset to zero in spring. Parallel to the vitamin D reduction, there was another significance reduction (p<0.05) of BMC from 3453.5 ± 339.4 to 3409.1 ± 278.0 g (Average ± DS) between autumn and winter.

## Conclusions

Our results agree with recently reported data (Halliday et al., 2011) confirming the supplying necessity at least during the winter to maintain adequate 25(OH)D3levels in elite soccer players. Our opinion is that the necessity of a possible supply must be taken into consideration trying to personalize the treatment at most, observing the fluctuations of 25(OH)D3 levels in each soccer player.

